# Associations between hearing loss and clinical outcomes: population-based cohort study

**DOI:** 10.1016/j.eclinm.2023.102068

**Published:** 2023-06-29

**Authors:** Marcello Tonelli, Natasha Wiebe, Meg Lunney, Maoliosa Donald, Tanis Howarth, Julie Evans, Scott W. Klarenbach, David Nicholas, Tiffany Boulton, Stephanie Thompson, Kara Schick Makaroff, Braden Manns, Brenda Hemmelgarn

**Affiliations:** a Department of Medicine, University of Calgary, Calgary, Canada; b Department of Medicine, University of Alberta, Edmonton, Canada; c Alberta Health Services, Calgary, Canada; d Alberta Health Services, Kitscoty, Canada; e Faculty of Social Work, University of Calgary, Calgary, Canada; f Department of Community Health Sciences, University of Calgary, Canada; g Faculty of Nursing, University of Alberta, Edmonton, Canada

**Keywords:** Hearing loss, Epidemiology

## Abstract

**Background:**

Hearing loss (HL) is a leading cause of disability worldwide, but its clinical consequences and population burden have been incompletely studied.

**Methods:**

We did a retrospective population-based cohort study of 4,724,646 adults residing in Alberta between April 1, 2004 and March 31, 2019, of whom 152,766 (3.2%) had HL identified using administrative health data. We used administrative data to identify comorbidity and clinical outcomes, including death, myocardial infarction, stroke/transient ischemic attack, depression, dementia, placement in long-term care (LTC), hospitalization, emergency visits, pressure ulcers, adverse drug events and falls. We used Weibull survival models (binary outcomes) and negative binomial models (rate outcomes) to compare the likelihood of outcomes in those with vs without HL. We calculated population-attributable fractions to estimate the number of binary outcomes associated with HL.

**Findings:**

The age-sex-standardized prevalence of all 31 comorbidities at baseline was higher among participants with HL than those without. Over median follow-up of 14.4 y and after adjustment for potential confounders at baseline, participants with HL had higher rates of days in hospital (rate ratio 1.65, 95% CI 1.39, 1.97), falls (RR 1.72, 95% CI 1.59, 1.86), adverse drug events (RR 1.40, 95% CI 1.35, 1.45), and emergency visits (RR 1.21, 95% CI 1.14, 1.28) compared to those without, and higher adjusted hazards of death, myocardial infarction, stroke/transient ischemic attack, depression, heart failure, dementia, pressure ulcers and LTC placement. The estimated number of people with HL who required new LTC placement annually in Canada was 15,631, of which 1023 were attributable to HL. Corresponding estimates for new dementia among people with HL were 14,959 and 4350, and for stroke/TIA the estimates were 11,582 and 2242.

**Interpretation:**

HL is common, is often accompanied by substantial comorbidity, and is associated with significant increases in risk for a broad range of adverse clinical outcomes, some of which are potentially preventable. This high population health burden suggests that increased and coordinated investment is needed to improve the care of people with HL.

**Funding:**

10.13039/501100000024Canadian Institutes of Health Research; David Freeze chair in health services research.

**Research in context**

**Evidence before this study**

What is the burden of comorbidity among community-dwelling people with hearing loss, and what is the excess risk of patient-important outcomes among people with this common chronic condition?

**Added value of this study**

This population-based study of >4 million people demonstrates that hearing loss is associated with a high burden of comorbidity as well as substantial increases in a broad range of adverse outcomes including cardiovascular events, hospitalization, placement in long-term care, emergency visits and adverse drug events.

**Implications of all the available evidence**

The high burden of serious illness among people with HL and the potential for early intervention to improve outcomes argue in favor of increased investment aimed at preventing HL while improving health care for those already affected.

## Introduction

Hearing loss (HL) affects nearly 1.6 billion people and is the third-leading cause of disability worldwide.^1^, ^2^, ^3^ Since the prevalence of HL increases with age, its global impact is expected to increase in parallel with population aging.^4^ HL is independently associated with increased mortality and the risk of incident dementia,^5^, ^6^, ^7^, ^8^ but less is known about the association between hearing loss and other patient-important outcomes such as cardiovascular disease, hospitalization, or placement in long-term care facilities. In addition, the burden of chronic disease and other comorbidity among people with hearing loss has been incompletely studied.^9^ Finally, although the potential for communication barriers between people with HL and their care providers is clear, whether people with HL are at excess risk of potentially preventable outcomes such as adverse drug events, pressure ulcers, or emergency department visits is less so. This information would be potentially useful for decision-makers who seek to understand the current and projected population health burden associated with HL, and may guide the design of interventions that aim to improve care for people with this condition.

We designed this population-based study of people treated in a universal health system to investigate the association between HL and a broad range of clinical outcomes. Important secondary objectives were to describe the burden of comorbidities observed among people with HL, and to identify clinical factors that might modify any association between HL and adverse outcomes.

## Methods

We reported this retrospective population-based cohort study according to the STROBE guidelines.^10^ The institutional review boards at the Universities of Alberta (Pro 00053469) and Calgary (REB16-1575) approved the study and waived the requirement for participants to provide consent.

### Data sources and cohort

We used an existing database, which incorporates patient registry, physician claims, hospitalizations, and ambulatory care utilization data from all adults registered with the provincial health ministry in Alberta, Canada; and links them with data from provincial clinical laboratories. This database has been widely used^11^, ^12^, ^13^ because of its population-based coverage of a geographically defined area, including demographic characteristics, health services utilization, and clinical outcomes. Additional information on the database is available elsewhere, including the validation of selected data elements.^14^ All Alberta residents are eligible for insurance coverage by Alberta Health with >99% registered as participants. The database was used to assemble cohorts of adults who resided in Alberta, Canada between April 1, 2004 and March 31, 2019. The index date was April 1, 2004, the day of first contact with Alberta Health, or the participants’ 18th birthday, whichever was latest.

### Hearing loss, comorbidities, and other characteristics

We created an algorithm to identify participants with HL using provider claims, at least two claims 30 days apart. This algorithm has not been validated but is based on those used in prior studies.^15^^,^^16^ We created a second algorithm for hearing loss using one hospitalization or two provider claims within 2 years. Eligible ICD-9, ICD-9-CM and ICD-10-CA codes are found in Supplemental Table S1.

We classified each participant with respect to the presence or absence of 31 chronic conditions at baseline (lookback extended as far as April 1994 where records were available).^17^

We defined 26 of these morbidities using a previously published framework of validated algorithms as applied to Canadian provider claims, hospitalizations, and ambulatory care data, each of which had positive predictive values ≥70% as compared to a gold standard measure such as chart review.^18^ Morbidities included atrial fibrillation, alcohol misuse, asthma, cancer (lymphoma, all metastatic cancers, non-metastatic breast, cervical, colorectal, pulmonary, and prostate cancer), chronic heart failure, chronic pain, chronic obstructive pulmonary disease, chronic liver disease (viral hepatitis B, cirrhosis), severe constipation, dementia, depression, diabetes, epilepsy, hypertension, hypothyroidism, inflammatory bowel disease, irritable bowel syndrome, multiple sclerosis, coronary artery disease (defined as myocardial infarction, coronary bypass artery graft, percutaneous coronary intervention), Parkinson's disease, peptic ulcer disease, peripheral artery disease, psoriasis, rheumatic diseases, schizophrenia and stroke or transient ischemic attack. Detailed methods for classifying comorbidity status and the specific algorithms used have been previously detailed.^18^ We defined gout, fragility fractures (wrist, forearm, spine, hip, humerus, and pelvis), and osteoporosis using similarly validated administrative algorithms.^19^^,^^20^ We defined severe obesity (body mass index [BMI] >35 kg/m^2^ before January 1, 2017 and BMI >40 kg/m^2^ after January 1, 2017) using a fee modifier as in our previous work.^21^ We defined severe chronic kidney disease by sustained estimated glomerular filtration rate (eGFR) < 30 mL/min per 1.73 m^2^ and/or registration with a provincial kidney replacement program.

As in our prior work, we used administrative data to identify age, biological sex, and rural residence location.^22^ We included the Pampalon index of material deprivation,^23^^,^^24^ which uses residential postal code to categorize participants into five categories of socioeconomic inequalities in health care services and population health with five representing the most deprived neighbourhoods.

### Outcomes

We assessed all-cause mortality, and the first occurrence during follow-up of myocardial infarction,^18^^,^^25^ stroke or transient ischemic attack,^18^^,^^26^ or depression episode.^18^^,^^27^ Depression episodes were allowed to occur at most every 2 years. We also assessed new (not present at baseline) heart failure,^18^^,^^27^ dementia,^18^^,^^27^ and long-term care placement. We also assessed length of hospital stays, number of emergency visits, adverse drug events,^28^ pressure ulcers^28^ and falls.^28^ Emergency visits were also categorized as “preventable”, “low acuity” and “other” according to work by Gruneir et al.^29^

### Statistical analyses

All analyses were done using Stata MP 17·0 (www.stata.com). We reported baseline descriptive statistics as counts and percentages, or means with standard deviations. We used time-to-event Weibull survival models to determine the hazard ratios of binary outcomes by hearing loss (HL) status (yes or no). We used negative binomial models to determine rate ratios of count outcomes by HL status, with an offset term of the natural logarithm for days of follow-up and modeled participants as a random intercept. We treated HL as a time-varying covariate, and thus events occurring any time within the study period prior to HL were included in the no-HL group and after hearing loss in the HL group. We divided participants into age-sex strata where age was grouped into 5-year intervals. In the Weibull modal, we modeled age-sex strata using shared frailty with a gamma distribution. In the negative binomial models, we estimated variances using the clustered sandwich estimator on the age-sex strata.

We adjusted for baseline or time-varying neighbourhood material deprivation quintile, rural residence and the 31 morbidities, in separate models. We determined that the proportional hazard assumption was satisfied by examining plots of the log-negative-log of within-group survivorship probabilities versus log-time for the Weibull models and we determined that the proportional rate assumption was satisfied by examining rate plots for the negative binomial models. We report the number, percentage or rate (per 100 patient-years) of events, and the age-sex adjusted, the baseline fully adjusted and time-varying fully adjusted hazard ratios (HR) or rate ratios (RR) with corresponding 95% confidence intervals (CI). Additionally we report the annual number of cases, population attributable fraction,^30^ and the annual number of cases attributable to HL for the binary outcomes in Alberta, using data from the time-varying fully adjusted models to estimate the population attributable fraction. We also estimated the annual number of cases and the annual number of cases attributable to HL in Canada, assuming that the results from Alberta were applicable to other Canadian provinces and using 2022 population data from StatCan.^31^ We deemed two-sided p values <0.05 to be statistically significant.

Missing values occurred in the following variables: residence location (13.3%), material deprivation quintile (14.6%), and obesity (26.9%). For the purposes of modelling, we used indicator variables to represent missingness.

### Sensitivity analyses

We explored whether certain characteristics (age categorized as 18–<50 y, 50–<70 y, and ≥70 y; biological sex; number of morbidities categorized as 0, 1–3 and ≥4) were potential modifiers of the association between hearing loss and four clinical outcomes with large effect sizes, using interaction terms. We also considered a second algorithm defining hearing loss.

### Role of funding

The study was supported by MT's David Freeze Chair in Health Services Research at the University of Calgary. The sponsors had no role in the design and conduct of the study; collection, management, analysis, and interpretation of the data; preparation, review, or approval of the manuscript; nor in the decision to submit the manuscript for publication.

## Results

### Cohort

**Fig. 1:**
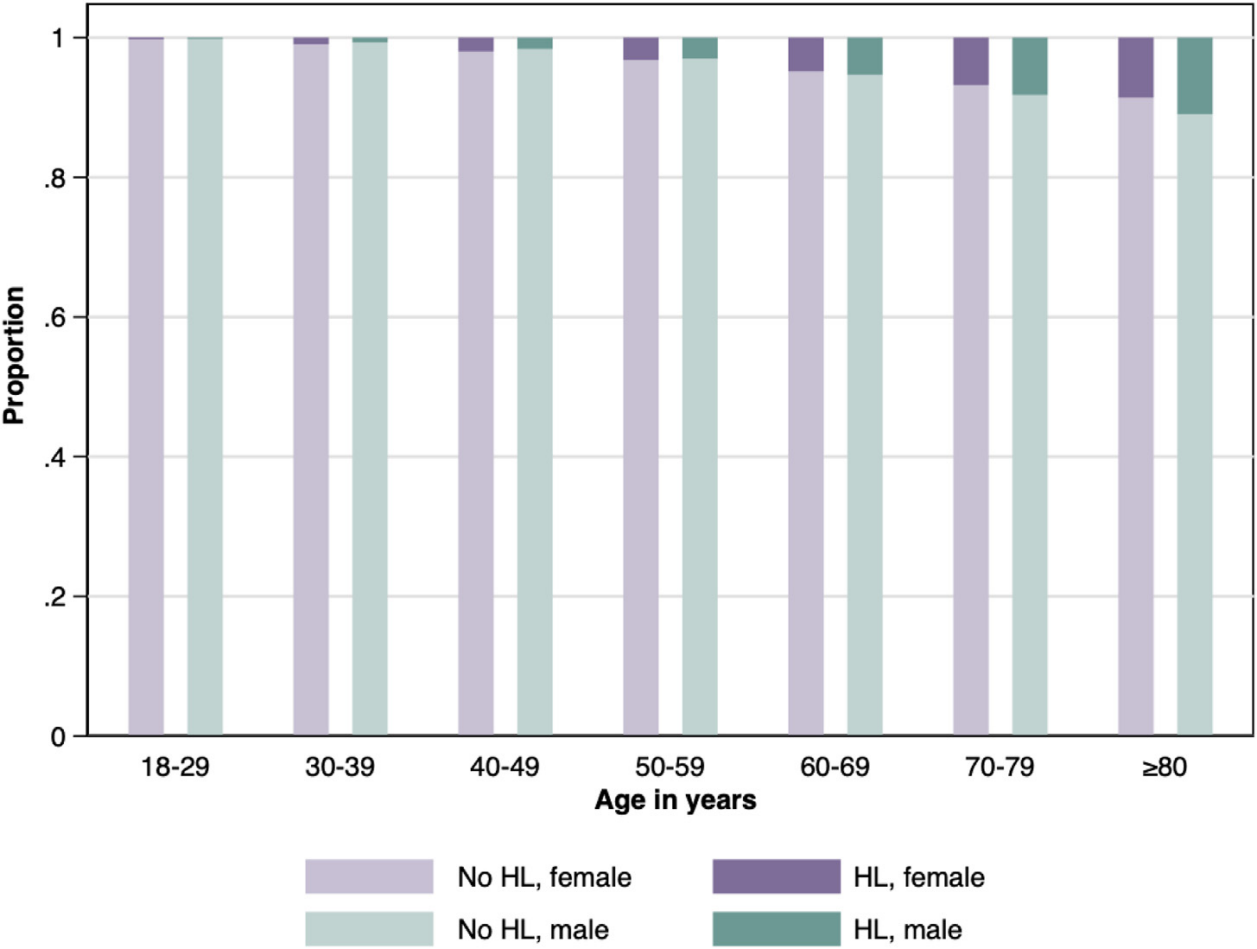
**Prevalence of hearing loss, by biological sex and age.** HL, Hearing loss. Each participant's age has been classified into one of 7 categories: 18–29, 30–39, 40–49, 50–59, 60–69, 70–70, and ≥80 years. The heights of the bars depict the percentage of participants with and without hearing loss by age group and biological sex.

We identified 4,724,646 adult participants during the study period, of whom 94% had at least one encounter during follow-up. At baseline, 78,172 (1.7%) had HL and 74,594 (1.6%) received a new HL diagnosis during follow-up, yielding a total of 152,766 (3.2%) participants with HL. As expected, the prevalence of HL increased with age (Fig. 1) and increased over time: from 2.4% in 2004 to 2.9% in 2018.

**Fig. 2:**
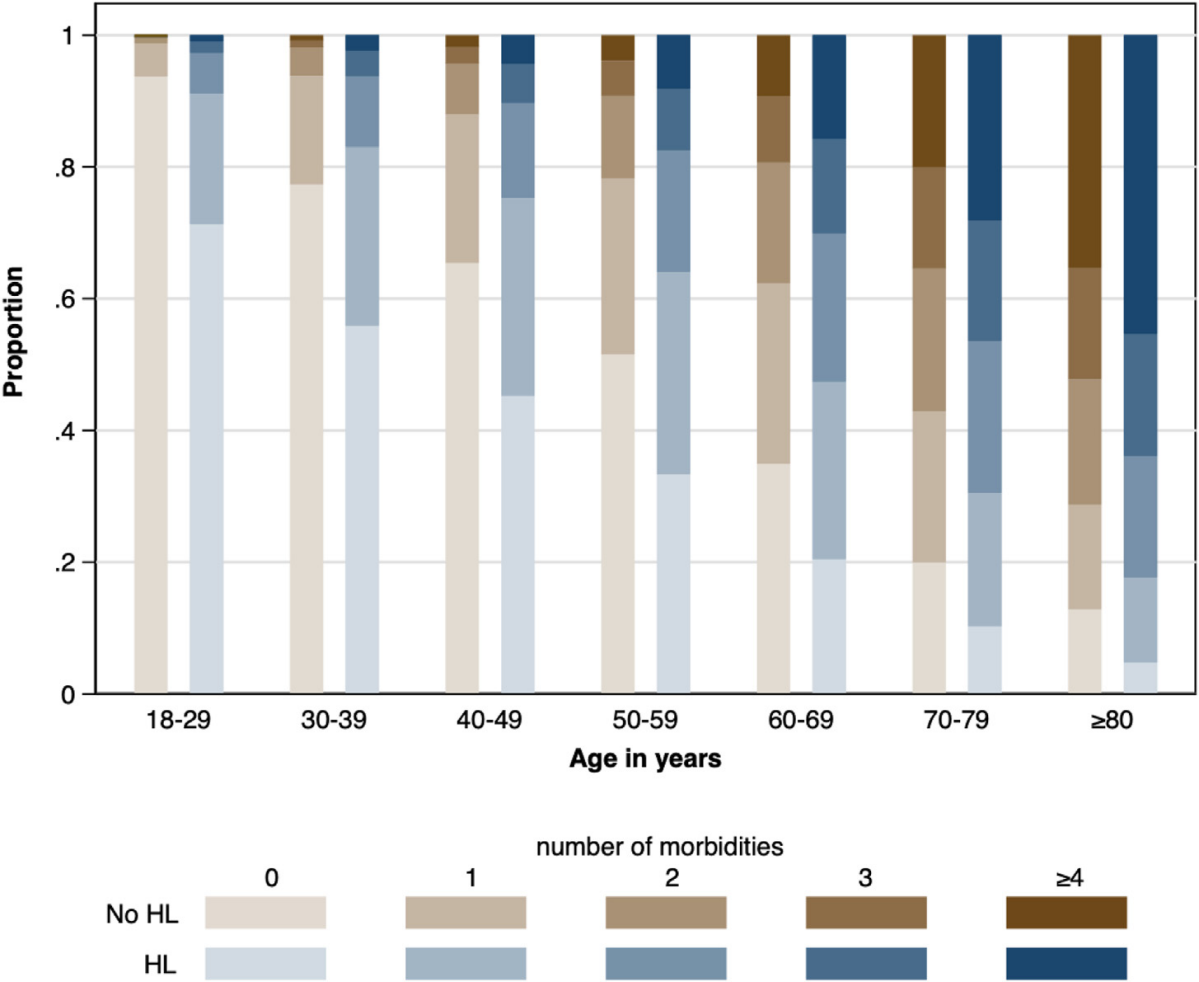
**Prevalence of comorbidity among people with and without hearing loss, by age**. HL, Hearing loss. Each participant's number of morbidities has been classified into one of 5 categories: none, 1 through 3, or 4 or more morbidities, and each participant's age has been classified into one of 7 groups: 18–29, 30–39, 40–49, 50–59, 60–69, 70–70, and ≥80 years. The heights of the bars depict the percentage of participants who fall into each morbidity category by hearing loss status and age group.

Table 1 shows the characteristics of the study population at baseline. For participants with HL, the mean age was 52.4 years and 50.0% were female compared to a mean age of 36.5 and 49% female for those without HL. The total number of comorbidities (Fig. 2) and the prevalence of each comorbidity (Table 1) were higher in participants with HL than those without. All comorbidities remained more prevalent in those with HL after standardization for age and sex. The most frequent morbidities for participants with HL were hypertension (26.8%), chronic pain (19.7%), obesity (13.2%), depression (10.8), and chronic pulmonary disease (9.6%). After standardization for age and sex, participants with HL were followed by more physicians (mean 4.1 vs 2.5) and more physician types (mean 2.3] vs 1.4) than those without, and were more likely to have a mental health condition (10.0% vs 5.0%, p < 0.001).

**Table 1:** Demographic and clinical characteristics at baseline by hearing loss status.

Characteristics	Not standardized for age-sex strata		Standardized for age-sex strata	
	Hearing loss	No hearing loss	Hearing loss	No hearing loss
N	152,766	4,571,880		
Age, y^a^	52.4 [17.5]	36.5 [17.1]	–	–
Male	76,453 (50.0)	2,332,302 (51.0)	–	–
Rural residence	15,041 (10.1)	413,313 (10.3)	13,554 (9.3)	414,005 (10.4)
Material deprivation^a^	3.1 [1.4]	3.2 [1.4]	3.1 [1.5]	3.2 [1.4]
Morbidities				
Obesity	18,333 (13.2)	338,895 (11.6)	16,383 (12.2)	340,470 (11.6)
Hypertension	40,959 (26.8)	408,808 (8.9)	32,704 (21.7)	418,317 (9.2)
Chronic pain	30,143 (19.7)	337,071 (7.4)	24,922 (16.5)	342,275 (7.5)
Depression	16,461 (10.8)	208,424 (4.6)	14,052 (9.3)	210,523 (4.6)
Diabetes	11,663 (7.6)	132,681 (2.9)	9669 (6.4)	135,233 (3.0)
Chronic pulmonary	14,686 (9.6)	138,431 (3.0)	11,647 (7.7)	141,100 (3.1)
Hypothyroid	10,997 (7.2)	113,098 (2.5)	8762 (5.8)	115,342 (2.5)
Osteoporosis	10,604 (6.9)	94,511 (2.1)	8241 (5.5)	96,571 (2.1)
Gout	7142 (4.7)	74,502 (1.6)	5721 (3.8)	76,221 (1.7)
Stroke/TIA	7165 (4.7)	59,398 (1.3)	5650 (3.7)	60,635 (1.3)
Fragility fracture	3956 (2.6)	49,223 (1.1)	3168 (2.1)	49,972 (1.1)
Cancer	4285 (2.8)	41,989 (0.9)	3452 (2.3)	42,858 (0.9)
Chronic heart failure	4746 (3.1)	46,889 (1.0)	3871 (2.6)	47,685 (1.0)
Alcohol misuse	2396 (1.6)	43,152 (0.9)	2072 (1.4)	43,573 (1.0)
Asthma	3801 (2.5)	42,472 (0.9)	3052 (2.0)	43,061 (0.9)
Atrial fibrillation	4108 (2.7)	35,914 (0.8)	3261 (2.2)	36,656 (0.8)
Coronary artery d.	4343 (2.8)	37,322 (0.8)	3350 (2.2)	38,337 (0.8)
Irritable bowel s.	3040 (2.0)	26,157 (0.6)	2374 (1.6)	26,625 (0.6)
Epilepsy	1801 (1.2)	22,860 (0.5)	1533 (1.0)	23,113 (0.5)
Rheumatic d.	2746 (1.8)	23,570 (0.5)	2109 (1.4)	24,094 (0.5)
Dementia	1703 (1.1)	19,662 (0.4)	1484 (1.0)	19,829 (0.4)
Schizophrenia	1150 (0.8)	18,571 (0.4)	973 (0.6)	18,786 (0.4)
Severe constipation	1118 (0.7)	10,310 (0.2)	917 (0.6)	10,456 (0.2)
Inflammatory bowel d.	1200 (0.8)	15,612 (0.3)	987 (0.7)	15,828 (0.3)
Multiple sclerosis	758 (0.5)	10,969 (0.2)	606 (0.4)	11,154 (0.2)
Peripheral arterial d.	1026 (0.7)	9240 (0.2)	800 (0.5)	9412 (0.2)
Psoriasis	649 (0.4)	7737 (0.2)	523 (0.3)	7886 (0.2)
Parkinson's d.	732 (0.5)	7812 (0.2)	583 (0.4)	7936 (0.2)
Severe CKD	360 (0.2)	4008 (0.1)	316 (0.2)	4043 (0.1)
Peptic ulcer d.	341 (0.2)	3443 (0.1)	277 (0.2)	3493 (0.1)
Liver d.	182 (0.1)	2995 (0.1)	163 (0.1)	3016 (0.1)
Complexity markers				
Number of morbidities^a^	1.3 [1.6]	0.4 [1.0]	1.0 [1.5]	0.5 [1.0]
Mental health condition	18,486 (11.8)	247,447 (5.3)	15,800 (10.4)	249,964 (5.4)
Number of prescriptions^a^	0.3 [3.4]	1.0 [6.0]	1.0 [6.1]	1.0 [5.9]
Types of physicians^a^	2.3 [1.7]	1.4 [1.3]	2.3 [1.7]	1.4 [1.3]
Number of physicians^a^	4.0 [3.7]	2.5 [3.0]	4.1 [3.7]	2.5 [3.0]

CKD, Chronic kidney disease; d, Disease; s, Syndrome; SD, Standard deviation; TIA, Transient ischemic attack.

N (%) or mean [SD] as appropriate. All characteristics were obtained at baseline. Participants in the group with hearing loss developed hearing loss at some point during follow-up. The age-sex strata are created using 5-year intervals of age. The differences between the groups were tested by regressing hearing loss onto each covariate in term, not weighted or weighted by age-sex strata, using logistic regression. All differences were significant at p < 0.001 except for the following: rural residence had a p = 0.007 for percentages that were not standardized for age-sex strata, and number of prescriptions had a p = 0.85 for means that were standardized for age-sex strata.

a Mean [SD].

### Clinical outcomes

During median follow-up of 14.4 years, 7.0% of participants (with or without hearing loss) died, 20.6% experienced an episode of depression, 4.7% experienced a stroke or a transient ischemic attack, 3.4% developed heart failure, 2.9% developed dementia, 1.4% had a myocardial infarction, and 1.3% were placed into longterm care (Table 2). Participants spent 78.9 days in hospital per 100 patient-years (100py). There were 41.9 emergency department visits per 100py, 0.37 falls per 100py, 0.33 adverse drug events per 100py, and 0.019 pressure ulcers per 100py.

**Table 2:** Clinical outcomes by hearing loss status.

Outcome	Hearing loss	No hearing loss	Age-sex adjusted (baseline)	Fully adjusted (baseline)	Fully adjusted (time-varying)
	Events (%)		(95% CI)		
All-cause mortality	25,206 (16.5)	304,391 (6.7)	**1.79 (1.76, 1.81)**	**1.25 (1.23, 1.27)**	1.00 (0.99, 1.02)
Acute MI	4904 (3.2)	63,283 (1.4)	**1.42 (1.38, 1.47)**	**1.10 (1.07, 1.13)**	1.03 (0.997, 1.06)
Stroke/TIA	18,248 (12.2)	201,441 (4.4)	**1.82 (1.80, 1.85)**	**1.37 (1.35, 1.39)**	**1.24 (1.22, 1.26)**
Depression	37,717 (27.6)	935,758 (20.4)	**2.10 (1.98, 2.22)**	**1.48 (1.40, 1.57)**	**1.16 (1.14, 1.17)**
New heart failure	13,667 (9.3)	145,881 (3.2)	**1.73 (1.70, 1.76)**	**1.27 (1.25, 1.29)**	**1.10 (1.08, 1.12)**
New dementia	15,177 (10.1)	122,619 (2.7)	**2.16 (2.12, 2.20)**	**1.61 (1.59, 1.64)**	**1.41 (1.38, 1.43)**
New LTC placement	7204 (4.7)	55,500 (1.2)	**1.91 (1.82, 2.00)**	**1.46 (1.39, 1.53)**	**1.07 (1.04, 1.10)**
	Events (rate/100py)		RR (95% CI)		

Days in hospital	3,152,557 (204.4)	37,802,674 (75.1)	**5.50 (3.50, 8.62)**	**1.65 (1.39, 1.97)**	**1.33 (1.16, 1.52)**
Emergency visits	1,025,983 (66.5)	20,711,479 (41.1)	**1.65 (1.51, 1.79)**	**1.21 (1.14, 1.28)**	**1.14 (1.11, 1.18)**
Potentially preventable	236,743 (15.3)	3,694,971 (7.3)	**2.47 (2.16, 2.81)**	**1.37 (1.29, 1.47)**	**1.12 (1.08, 1.17)**
Low acuity	66,029 (4.3)	1,001,485 (2.0)	**2.79 (2.43, 3.19)**	**1.63 (1.51, 1.75)**	**1.15 (1.09, 1.22)**
Other	723,211 (46.9)	16,015,023 (31.8)	**1.42 (1.33, 1.51)**	**1.09 (1.05, 1.13)**	**1.11 (1.08, 1.15)**
Adverse drug events	12,080 (0.8)	157,941 (0.3)	**3.05 (2.52, 3.70)**	**1.40 (1.35, 1.45)**	**1.08 (1.04, 1.12)**
Pressure ulcer	716 (0.05)	9244 (0.02)	1.14 (0.89, 1.38)	**1.39 (1.21, 1.60)**	0.97 (0.85, 1.11)
Fall	17,252 (1.1)	173,942 (0.3)	**4.20 (3.27, 5.40)**	**1.72 (1.59, 1.86)**	**1.28 (1.21, 1.35)**

MI, Myocardial infarction; HR, Hazard ratio; LTC, Long-term care; PY, Patient-year; RR Rate ratio; TIA, Transient ischemic attack.

All models treat HL as a time-varying covariate, and thus events occurring any time within the study period prior to HL were included in the no-HL group and after hearing loss in the HL group.

We divided participants into age-sex strata where age was grouped into 5-year intervals. In the Weibull model, HR or RR (95% CI) are reported as appropriate. All-cause mortality, AMI, stroke/TIA, new HF (in those without heart failure), new dementia (in those without dementia) and new LTC placement (in those not in care) were regressed on time-varying hearing loss using Weibull regression with shared frailty modelled in 5-year age and sex groups. Length of hospital stays, ED visits, ADEs, pressure ulcers, and falls were regressed on time-varying hearing loss using negative binomial regression where the clustered sandwich estimator was used for variance estimation in the 5-year age and sex groups.

The other two models were further adjusted for rural residence status, material deprivation quintiles, obesity, hypertension, chronic pain, depression, chronic pulmonary disease, diabetes mellitus, hypothyroidism, osteoporosis, gout, stroke or TIA, fragility fractures, heart failure, cancer, asthma, alcohol misuse, coronary artery disease, atrial fibrillation, irritable bowel syndrome, rheumatic diseases, epilepsy, dementia, schizophrenia, inflammatory bowel disease, multiple sclerosis, severe constipation, peripheral artery disease, Parkinson's disease, psoriasis, severe chronic kidney disease, peptic ulcer disease, and chronic liver disease.

The adjusted (baseline) model used these covariates as assessed at baseline, whereas the adjusted (time-varying) model updated the covariates throughout follow-up.

Bold values indicate statistically significant at p < 0.05.

After adjustment for age and sex, participants with HL experienced substantially more adverse clinical outcomes than participants without HL (Table 2). The rate of hospitalization (total days) was 5.50 times higher (95% CI 3.50, 8.62), the rate of falls was 4.20 times higher (95% CI 3.27, 5.40), and of adverse drug events 3.05 times higher (95% CI 2.52, 3.70). Overall, the rate of emergency department visits was 1.65 times higher (95% CI 1.51, 1.79) in people with HL than without; the rates of low acuity and potentially preventable visits were both increased.

In fully adjusted models using baseline covariates, the risk of all-cause mortality, acute MI, stroke/TIA, depression, new heart failure, new dementia and new placement in LTC were all significantly more common in people with HL than those without. The magnitude of the excess risk ranged from hazard ratio (HR) 1.10 (95% CI 1.07, 1.13) for acute MI to rate ratio (RR) 1.72 (95% CI 1.59, 1.86) for falls. After adjustment for time-varying covariates, the magnitude of the excess risk or rate associated with HL was attenuated for all outcomes, and remained significant for the majority, although not for the risk of mortality, the risk of acute MI, or the rate of pressure ulcers. The second algorithm defining hearing loss had similar results (Supplemental Table S2).

### Effect modification

We selected the clinical outcomes with the highest relative increases in events (risk of dementia, rate of hospital days, rate of falls, and risk of stroke or TIA) to explore age, sex, and number of morbidities as potential modifiers of the associations between HL and outcomes.

Age did not modify the association between HL and the rate of hospital days or the rate of falls, but significantly modified the associations between HL and the risk of dementia and the risk of stroke/TIA (p < 0.001), for which the magnitude of the excess risk associated with HL was greater for younger participants than older participants (Table 3). Biological sex modified the association between HL and all four outcomes: the magnitude of the excess risk associated with HL was greater in men than women for dementia, days in hospital and stroke/TIA (all p ≤ 0.002) but greater in women than men for falls (p = 0.01). The number of comorbidities also modified the association between HL and all four outcomes; the magnitude of the excess risk associated with HL was greater for participants with fewer comorbidities than those with more comorbidities (all p < 0.001).

**Table 3:** Clinical outcomes by hearing loss status.

Interaction	Stroke/TIA	New dementia	Days in hospital	Fall
	HR (95% CI)		RR (95% CI)	
Age, y	**<0.001**	**<0.001**	0.98	0.14
18–<50	**1.82 (1.71, 1.93)**	**2.57 (2.23, 2.96)**	0.96 (0.64, 1.45)	1.12 (0.90, 1.38)
50–<70	**1.17 (1.13, 1.20)**	**1.45 (1.37, 1.52)**	**1.09 (1.06, 1.12)**	1.01 (0.91, 1.12)
≥70	**1.03 (1.01, 1.05)**	**1.10 (1.08, 1.12)**	0.95 (0.88, 1.04)	1.01 (0.94, 1.08)
Sex	**0.002**	**<0.001**	**<0.001**	**0.01**
Female	**1.21 (1.19, 1.24)**	**1.31 (1.28, 1.35)**	1.03 (0.88, 1.21)	**1.40 (1.29, 1.51)**
Male	**1.27 (1.25, 1.30)**	**1.54 (1.50, 1.58)**	**1.87 (1.61, 2.18)**	**1.16 (1.07, 1.27)**
Number of morbidities	**<0.001**	**<0.001**	**<0.001**	**<0.001**
0	**2.24 (2.12, 2.38)**	**3.57 (3.23, 3.94)**	**1.33 (1.02, 1.73)**	**1.58 (1.33, 1.87)**
1–3	**1.36 (1.33, 1.39)**	**1.80 (1.75, 1.85)**	**1.39 (1.21, 1.59)**	**1.43 (1.33, 1.53)**
≥4	**1.06 (1.04, 1.08)**	**1.18 (1.15, 1.20)**	**1.16 (1.09, 1.23)**	**1.11 (1.06, 1.16)**

HR, Hazard ratio; RR, Rate ratio; TIA, Transient ischemic attack.

All models treat HL as a time-varying covariate, and thus events occurring any time within the study period prior to HL were included in the no-HL group and after hearing loss in the HL group.

HR or RR (95% CI) are reported as appropriate. Stroke/TIA and new dementia (in those without dementia) were regressed on time-varying hearing loss using Weibull regression and interacted with one of five potential modifiers (age categorized as 18–<50, 50–<70, and ≥70 years, sex, number of comorbidities categorized as 0, 1–3, or ≥4 morbidities) with shared frailty modelled in either 5-year age and sex groups, or simply age or sex groups, depending on the modifier. Length of hospital stays and falls were regressed on time-varying hearing loss using negative binomial regression and interacted with one of five potential modifiers where the clustered sandwich estimator was used for variance estimation in the 5-year age and sex groups, just age, or just sex groups.

The models were further adjusted for time-varying rural status, material deprivation quintiles, obesity, hypertension, chronic pain, depression, chronic pulmonary disease, diabetes mellitus, hypothyroidism, osteoporosis, gout, stroke or TIA, fragility fractures, heart failure, cancer, asthma, alcohol misuse, coronary artery disease, atrial fibrillation, irritable bowel syndrome, rheumatic diseases, epilepsy, dementia, schizophrenia, inflammatory bowel disease, multiple sclerosis, severe constipation, peripheral artery disease, Parkinson's disease, psoriasis, severe chronic kidney disease, peptic ulcer disease, and chronic liver disease.

Bold values indicate statistically significant at p < 0.05.

### Population attributable risk

The population attributable risk associated with HL for the binary outcomes in our dataset ranged from 0.3% for new LTC placement to 3.8% for depression (Supplemental Table S3).

After extrapolation to the entire Canadian adult population, the estimated number of people with HL who require new LTC placement annually in Canada was 15,631, of which 1023 were attributable to HL. Corresponding estimates for new dementia were 14,959 and 4350 and for stroke/TIA the estimates were 11,582 and 2242.

## Discussion

This population-based cohort study of more than 4 million adults treated in a universal health system had four key findings. First, even after adjustment for age and sex, HL is associated with a substantially increased burden of comorbidity. Second, HL is associated with excess risk for a broad range of adverse clinical outcomes, including substantial increases in the rates of potentially preventable outcomes such as hospitalization, LTC placement, emergency visits, adverse drug events and falls. Third, progressively more thorough adjustment for comorbidities at baseline and during follow-up tended to attenuate but not eliminate the excess risk associated with HL. Fourth, the magnitude of the excess risk associated with HL appeared to be higher in people who were otherwise at lower risk, such as those who were younger or had less comorbidity.

Previous work has identified HL as a strong risk factor for dementia^7^^,^^32^ and falls.^33^, ^34^, ^35^ HL has also been associated with above-average rates of all-cause mortality^6^^,^^7^ and hospitalization,^36^ an increased prevalence of comorbidity,^37^ and perhaps an excess risk of cardiovascular events.^38^^,^^39^ Prior research clearly shows that HL is common among people in LTC facilities, and one study suggests that among people receiving homecare, the presence of HL seems to hasten placement in a LTC facility.^40^ Studies examining the link between HL and emergency room visits,^41^ adverse drug reactions or pressure ulcers are less conclusive or have not been done. Although these prior studies were of high quality, they have typically included a much smaller sample size, could not adjust for comorbidity to the same extent, and were often done in populations without access to universal healthcare. Our study extends prior work by including a broad range of clinical outcomes over an extended follow-up period in a large population-based cohort, reducing the possibility of selection bias, ensuring complete capture of clinical outcomes during follow-up, and allowing us to estimate population attributable risk.

Why would HL be associated with excess risk? For outcomes such as adverse drug events, hospitalization and preventable emergency visits, we speculate that communication barriers between health professionals and patients are responsible. The independently increased risk of LTC placement warrants further investigation, since it might suggest that additional supports are required for people with HL to continue living independently despite their disability. The excess risk of falls may be due to increased cognitive load, reduced environmental awareness, or perhaps concomitant proprioceptive/vestibular dysfunction.^34^^,^^42^ For other outcomes such as stroke, the apparently increased risk may relate to residual confounding by comorbidities that are also associated with HL, such as hypertension or vascular disease. However, even for these outcomes it is plausible that people with HL do not always receive optimal treatment for these comorbidities due to communication challenges, provider bias,^43^^,^^44^ depression, or other barriers to care, which in turn warrants critical reflection about how best to improve equity and accessibility to health care services for people with HL. Even if some of the apparent excess risk is due to residual confounding by comorbidity, our data indicate that the presence of HL may be clinically useful as a marker for high risk of adverse outcomes.

The finding that the excess risk of adverse outcomes associated with HL was greater in participants who would otherwise be at lower risk due to younger age or fewer comorbidities not been previously reported to our knowledge. This effect modification was independent of multiple potential confounders, and may be due to the absence of competing risk factors in these healthier subgroups, although this explanation is speculative.

Our results together with the prior literature on this topic have two potential implications. First, research on the causes, consequences and optimal treatment of hearing loss is significantly underfunded. For example, in 2017 only 0.6% of the total National Institutes of Health budget was committed to grants on hearing loss research.^45^^,^^46^ This level of funding is not commensurate with the global burden of hearing loss, and additional investment is required. Second, the findings from this observational study do not demonstrate a causal link between HL and adverse outcomes. However, our results demonstrate that people with HL constitute a high-risk group that should arguably be prioritized for intervention, especially given the potentially preventable nature of many of the outcomes that we studied. The relatively large number of such outcomes that occur in people with HL (as well as the smaller but still substantial number that are attributable to HL) indicate that action is required in three broad areas: better care for people with HL who experience medical illness, including support for communication as well as management of comorbidity; better treatment and rehabilitation of HL specifically; and more effective public health actions to prevent HL.^47^ Our findings support the need for coordinated national strategies for ear and hearing care,^48^ with input from a broad range of stakeholders. Such strategies are widely available for conditions such as cancer or mental illness -- but not for ear and hearing care, which is a missed opportunity that must be addressed.

Our study has several important strengths, including the use of population-based data from a geographically defined area served by a universal health care system; large sample size and adequate follow-up time; use of validated algorithms for ascertaining the presence or absence of comorbidity; rigorous analytical methods; and inclusion of a broad range of clinically relevant and patient-important outcomes. However, our study also has limitations that should be considered when interpreting results. First, like all studies using administrative data, residual confounding is possible by unmeasured characteristics such as smoking, noise exposure, physical activity, diet, access to assistive devices such as hearing aids, or the availability of social supports. Second, although the large majority (94%) of participants had at least one encounter during follow-up, those with more encounters may have been more likely to be classified with HL and so ascertainment bias could have exaggerated the association between HL and adverse outcomes. Third, although we based our algorithm for HL on published studies using administrative data, it has not been validated against an independent standard such as audiological testing. To the extent that claims data may detect only more severe cases of HL, our findings likely underestimate the population health burden of HL. Third, the absolute effect size for some associations was statistically significant but clinically small. Finally, we studied people from a single Canadian province and our findings will require validation in other settings.

In conclusion, HL is a common condition that is associated with a high burden of comorbidity and substantial increases in a broad range of potentially preventable adverse outcomes including hospitalization, LTC placement, emergency visits, adverse drug events and falls. The high burden of serious medical illness among people with HL, the potential for early intervention to improve outcomes, and the relatively large absolute attributable burden of HL all argue in favor of increased investment aimed at preventing HL while improving health care for those already affected.

## Contributions

MT conceived the study. NW did the statistical analyses. MT and NW wrote the first draft of the manuscript. All authors contributed to the design, interpretation of results and critical revision of the article for intellectually important content. MT and NW had full access to all the data in the study and takes responsibility for the integrity of the data and the accuracy of the data analysis.

## Data sharing statement

We cannot make our dataset available to other researchers due to our contractual arrangements with the provincial health ministry (Alberta Health), who is the data custodian. Researchers may make requests to obtain a similar dataset at https://absporu.ca/research-services/service-application/.

## Declaration of interests

TH is a (non-paid) board director for the Canadian Academy of Audiology. SWK is a director of the Real World Evidence Consortium, and the Alberta Drug and Therapeutic Evaluation Consortium (Universities of Alberta, Calgary, and Institute of Health Economics); these entities receive funding from decision makers and industry to conduct research. All research funding is made to the academic institution; investigator retains full rights of academic freedom and right to publish. This relationship is not related to the current work. Otherwise there were no potential conflicts of interest to declare.
