# Supplementary material for: Associations between hearing loss and clinical outcomes: population-based cohort study
Source: eClinicalMedicine. 2023 Jun 29;61:102068. doi: 10.1016/j.eclinm.2023.102068 (PMC10331811; doi:10.1016/j.eclinm.2023.102068)
Supplement: Supplemental Tables S1–S3 [file mmc1.docx]

**Supplement for:**

**Associations between hearing loss and clinical outcomes: population-based cohort study**

Marcello Tonelli MD SM MSc, Department of Medicine, University of Calgary, Calgary

Natasha Wiebe MMath PStat, Department of Medicine, University of Alberta, Edmonton

Meg Lunney PhD, Department of Medicine, University of Calgary, Calgary

Maoliosa Donald PhD BScPT, Department of Medicine, University of Calgary, Calgary

Tanis Howarth MSc RAud, Alberta Health Services, Calgary

Julie Evans MSc RSLP, Alberta Health Services, Kitscoty

Scott W Klarenbach MD MSc, Department of Medicine, University of Alberta, Edmonton

David Nicholas PhD RSW, Faculty of Social Work, University of Calgary, Calgary

Tiffany Boulton PhD, Department of Community Health Sciences, University of Calgary

Stephanie Thompson MD PhD, Department of Medicine, University of Alberta, Edmonton

Kara Schick Makaroff PhD RN, Faculty of Nursing, University of Alberta, Edmonton

Braden Manns MD MSc, Department of Medicine, University of Calgary, Calgary

Brenda Hemmelgarn PhD MD, Department of Medicine, University of Alberta, Edmonton

**Supplemental Table S1. Administrative codes for hearing loss**

**Supplemental Table S2. Clinical outcomes by hearing loss status – alternative definition for hearing loss**

**Supplemental Table S3. Projected population burden and population attributable fraction for Alberta and Canada**

**Supplemental Table S1. Administrative codes for hearing loss**

| **ICD-9** | **ICD-9-CM** | **ICD-10-CA** | **Description** |
| --- | --- | --- | --- |
| - | 388.01 | H91.1 | Presbycusis |
| 388.1 | 388.1 | H83.3 | Noise effects on inner ear |
| 388.2 | 388.2 | H91.2 | Sudden hearing loss, unspecified |
| 389 | 389 | H90, H91.0, H91.3-H91.9 | Hearing loss |
| V41.2 | V41.2 | - | Problems with hearing |

**Supplemental Table S2. Clinical outcomes by hearing loss status – alternative definition for hearing loss**

| **Outcome** | **Primary** | **Sensitivity** |
| --- | --- | --- |
|  | HR (95%CI) | |
| All-cause mortality | 1.00 (0.99,1.02) | **1.07 (1.06,1.08)** |
| Acute MI | 1.03 (0.997,1.06) | 1.03 (0.999,1.05) |
| Stroke/TIA | **1.24 (1.22,1.26)** | **1.20 (1.18,1.22)** |
| Depression | **1.16 (1.14,1.17)** | **1.16 (1.15,1.18)** |
| New heart failure | **1.10 (1.08,1.12)** | **1.09 (1.07,1.10)** |
| New dementia | **1.41 (1.38,1.43)** | **1.32 (1.30,1.34)** |
| New LTC placement | **1.07 (1.04,1.10)** | **1.06 (1.04,1.08)** |
|  | RR (95%CI) | |
| Days in hospital | **1.33 (1.16,1.52)** | **1.23 (1.08,1.41)** |
| Emergency visits | **1.14 (1.11,1.18)** | **1.08 (1.04,1.12)** |
| Potentially preventable | **1.12 (1.08,1.17)** | **1.06 (1.01,1.11)** |
| Low acuity | **1.15 (1.09,1.22)** | **1.13 (1.06,1.20)** |
| Other | **1.11 (1.08,1.15)** | **1.04 (1.01,1.08)** |
| Adverse drug events | **1.08 (1.04,1.12)** | **1.10 (1.06,1.15)** |
| Pressure ulcer | 0.97 (0.85,1.11) | 1.02 (0.90,1.15) |
| Fall | **1.28 (1.21,1.35)** | **1.24 (1.19,1.30)** |

MI myocardial infarction, HR hazard ratio, LTC long-term care, RR rate ratio, TIA transient ischemic attack

All models treat HL as a time-varying covariate, and thus events occurring any time within the study period prior to HL were included in the no-HL group and after hearing loss in the HL group.

We divided participants into age-sex strata where age was grouped into 5-year intervals. In the Weibull model, HR or RR (95%CI) are reported as appropriate. All-cause mortality, AMI, stroke/TIA, new HF (in those without heart failure), new dementia (in those without dementia) and new LTC placement (in those not in care) were regressed on time-varying hearing loss using Weibull regression with shared frailty modelled in 5-year age and sex groups. Length of hospital stays, ED visits, ADEs, pressure ulcers, and falls were regressed on time-varying hearing loss using negative binomial regression where the clustered sandwich estimator was used for variance estimation in the 5-year age and sex groups.

The models were also adjusted for rural residence status, material deprivation quintiles, obesity, hypertension, chronic pain, depression, chronic pulmonary disease, diabetes mellitus, hypothyroidism, osteoporosis, gout, stroke or TIA, fragility fractures, heart failure, cancer, asthma, alcohol misuse, coronary artery disease, atrial fibrillation, irritable bowel syndrome, rheumatic diseases, epilepsy, dementia, schizophrenia, inflammatory bowel disease, multiple sclerosis, severe constipation, peripheral artery disease, Parkinson’s disease, psoriasis, severe chronic kidney disease, peptic ulcer disease, and chronic liver disease. The covariates were assessed at baseline and updated (time-varying) throughout follow-up.

**Supplemental Table S3. Projected population burden and population attributable fraction for Alberta and Canada**

| **Outcome** | **Risk of hearing loss in cases** | **Number of annual cases in people with HL (Alberta)** | **Number of annual cases in people with HL (Canada)** | **Population attributable fraction** | **Number of annual cases attributable to HL**  **(Alberta)** | **Number of annual cases attributable to HL**  **(Canada)** |
| --- | --- | --- | --- | --- | --- | --- |
| Stroke/TIA | 8.31% | 1,299 | 11,582 | 1.6% | 251 | 2,242 |
| Depression | 3.87% | 661 | 5,897 | 0.5% | 91 | 813 |
| New heart failure | 8.57% | 1,269 | 11,319 | 0.8% | 115 | 1,029 |
| New dementia | 11.01% | 1,677 | 14,959 | 3.2% | 488 | 4,350 |
| New LTC placement | 11.49% | 1,752 | 15,631 | 0.8% | 115 | 1,023 |

HL hearing loss, LTC long-term care, TIA transient ischemic attack

The number of annual cases (for stroke/TIA, depression, new heart failure, new dementia, or new LTC placement) in people with HL in Alberta was calculated from the study cohort. Albertans comprise about 11.2% of the Canadian population; we multiplied Alberta results by 8.91939 to approximate the mean number of cases per year in adult Canadians with HL.^31^ The population attributable fractions were calculated according to Mansournia and Altman.^30^ For example, the risk of HL in Albertans with stroke was 8.31% and the hazard ratio was 1.24. The PAF was 0.0831*(1-1/1.24)=0.016 or 1.6%. The number of annual cases attributable to HL in Alberta (or Canada) was the annual number of people with HL (at risk for the outcome) in Alberta (or Canada) multiplied by the population attributable fraction. The annual number of people with HL can be calculated from the total follow-up time (before the first event) in participants with HL divided by the mean follow-up time (before the first event) in participants with HL (e.g., 1,430,717/9.6=15,626 people with HL). Thus, in this example, the number of annual strokes attributable to HL in Alberta is 15,626*0.016=251.
