# Supplementary material for: Associations between hearing loss and clinical outcomes: population-based cohort study
Source: eClinicalMedicine. 2023 Jun 29;61:102068. doi: 10.1016/j.eclinm.2023.102068 (PMC10331811; doi:10.1016/j.eclinm.2023.102068)
Supplement: Figures S1–S12 [file mmc2.docx]

**Figure 1. Proportional hazards plot for all-cause mortality by hearing loss**

**
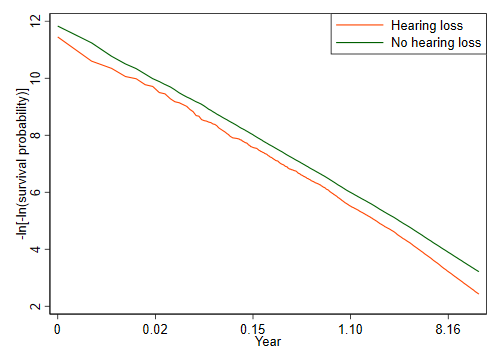
**

The log-negative-log within-group survivorship probabilities are sufficiently parallel across follow-up.

**Figure 2. Proportional hazards plot for acute myocardial infarction by hearing loss**


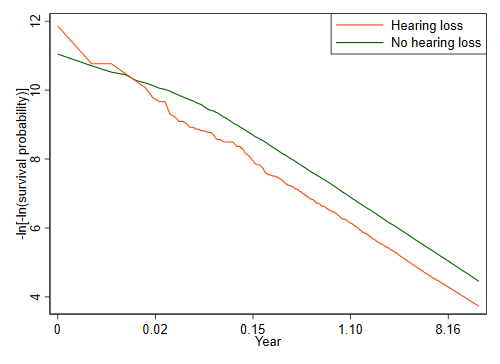


The log-negative-log within-group survivorship probabilities are sufficiently parallel across follow-up.

**Figure 3. Proportional hazards plot for stroke or transient ischemic attack by hearing loss**


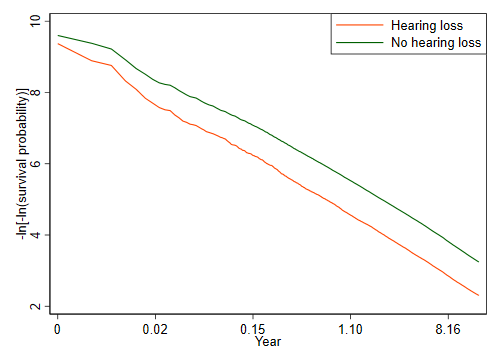


The log-negative-log within-group survivorship probabilities are sufficiently parallel across follow-up.

**Figure 4. Proportional hazards plot for depression by hearing loss**

**
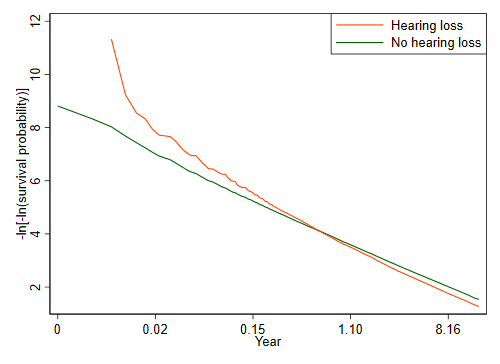
**

The log-negative-log within-group survivorship probabilities are sufficiently parallel across follow-up.

**Figure 5. Proportional hazards plot for new heart failure by hearing loss**

**
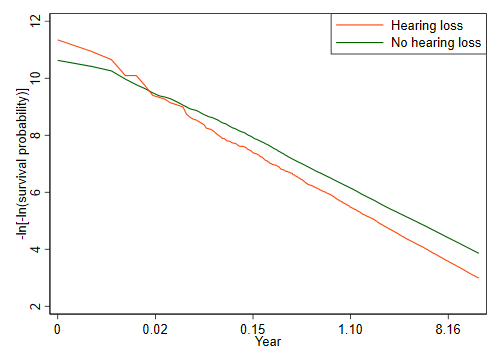
**

The log-negative-log within-group survivorship probabilities are sufficiently parallel across follow-up.

**Figure 6. Proportional hazards plot for new dementia by hearing loss**

**
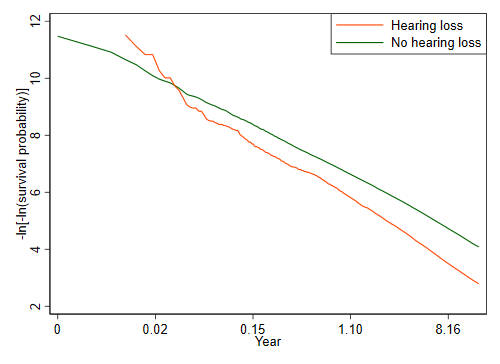
**

The log-negative-log within-group survivorship probabilities are sufficiently parallel across follow-up.

**Figure 7. Proportional hazards plot for new long-term care placement by hearing loss**

**
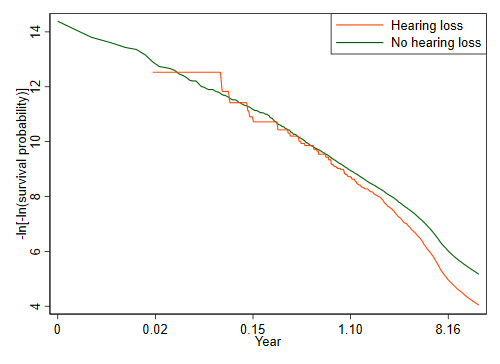
**

The log-negative-log within-group survivorship probabilities are sufficiently parallel across follow-up.

**Figure 8. Rate constancy plot for days in hospital by hearing loss**

**
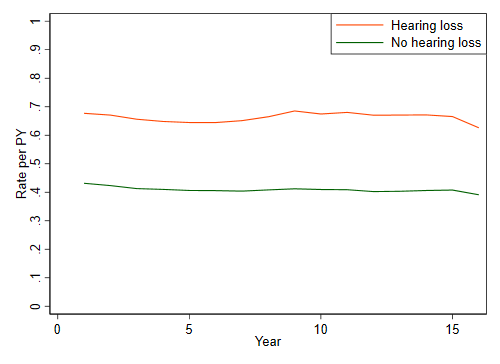
**

The rates are sufficiently constant across follow-up. PY participant-year

**Figure 9. Rate constancy plot for emergency visits by hearing loss**

**
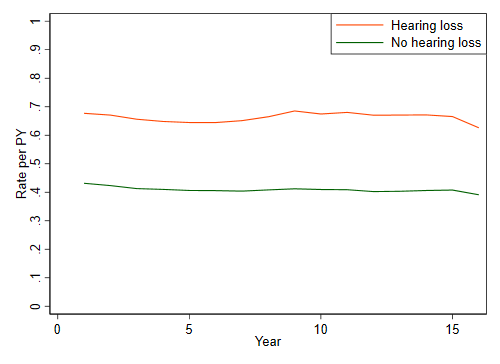
**

The rates are sufficiently constant across follow-up. PY participant-year

**Figure 10. Rate constancy plot for adverse drug events by hearing loss**

**
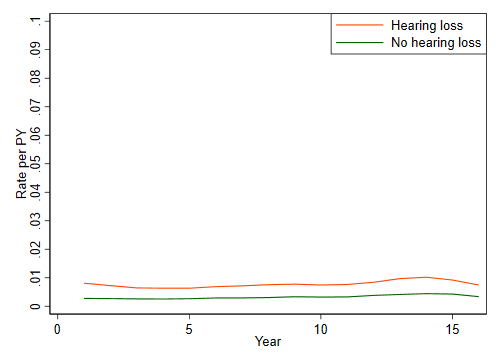
**

The rates are sufficiently constant across follow-up. PY participant-year

**Figure 11. Rate constancy plot for pressure ulcers by hearing loss**

**
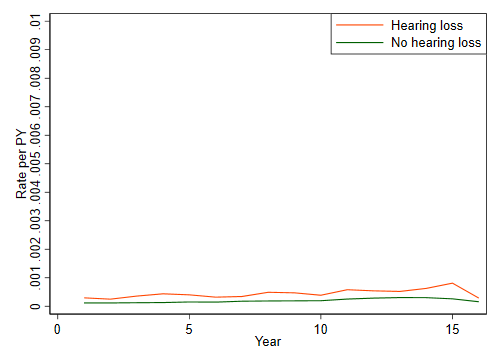
**

The rates are sufficiently constant across follow-up. PY participant-year

**Figure 12. Rate constancy plot for falls by hearing loss**

**
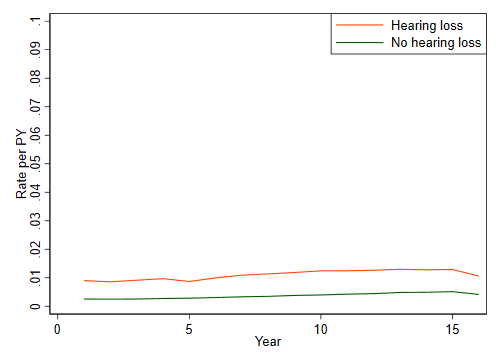
**

The rates are sufficiently constant across follow-up. PY participant-year
